# Rappels vaccinaux hors programme élargi de vaccination dans deux écoles de l’éducation de base de Yaoundé, Cameroun

**Published:** 2011-10-12

**Authors:** Clémence Vougmo Meguejio Njua, Félicitée Nguefack, David Chelo, Mathurin Tejiokem, Innocent Kago, Marie Kobela

**Affiliations:** 1 Centre Mère et Enfant (CME) de la Fondation Chantal BIYA de Yaoundé, Cameroun; 2 Faculté de Médecine et des Sciences Biomédicales - Université de Yaoundé I, Cameroun; 3 Centre Pasteur du Cameroun, Yaoundé, Cameroun; 4 Secrétariat Permanent du Programme Elargi de Vaccination, Yaoundé, Cameroun

**Keywords:** Vaccination, Programme Elargi de Vaccination, enfant, rappel, Cameroon

## Abstract

**Introduction:**

L'absence des rappels vaccinaux est problématique dans les pays en développement où certaines maladies évitables par la vaccination font encore des victimes chez les enfants en raison d'une immunisation incomplète. L'identification des raisons de non rappel vaccinaux permettrait de proposer des solutions adaptées afin d'améliorer le statut vaccinal des enfants au-delà de 12 mois.

**Méthodes:**

Cette étude descriptive transversale avait pour objectif d’évaluer le taux de rappels vaccinaux des enfants au-delà de la cible du programme élargi de vaccination (PEV). Elle s'est déroulée de Septembre à Novembre 2009 dans deux écoles d’éducation de base de Yaoundé. L’échantillonnage était consécutif et limité aux élèves âgés de deux à sept ans possédant des carnets de vaccination. Par souci d'uniformité, nous n'avons pas analysé les rappels des vaccins contre l'hépatite B et l'haemophilus introduits tardivement au Cameroun.

**Résultats:**

L’étude a porté sur 310 élèves. La tranche d’âge de 2 à 4 ans représentait 69%. Seul 223 enfants (71,9%) étaient correctement vaccinés. Quant aux rappels vaccinaux diphtérie-tétanos-coqueluche et poliomyélite, les couvertures étaient insignifiantes (2,7% et 0% respectivement pour la première et la deuxième dose). Les raisons évoquées étaient l'absence de sensibilisation des parents (50%), le prix élevé des vaccins (48,69%) et la désinformation (1,31%).

**Conclusion:**

Le recyclage du personnel de santé sur la vaccination est une nécessité. Les parents doivent être informés sur le déroulement, les prix et les lieux des rappels vaccinaux. La réduction des prix de vaccins faciliterait l'accès à une large tranche de la population.

## Introduction

La vaccination représente l'un des plus beaux succès de la santé publique au 20^e^ siècle. Elle a permis de sauver des millions de vies d'enfants depuis la mise en en æuvre des programmes nationaux de vaccination (PEV). Selon les estimations de l'OMS, la vaccination a prévenu environ 2,9 millions de décès par la rougeole, le tétanos néonatal, la coqueluche, et la poliomyélite en 1992 [1]. Cependant, son bénéfice s'estompe au fil du temps en l'absence de rappels vaccinaux [2]. Ces rappels visent à renforcer l'immunité déclinante, à prolonger la protection de l'enfant, et à interrompre la transmission de la maladie.

En France, une enquête sérologique a mis en évidence une perte de l'immunité vis-à-vis de la diphtérie avec l’âge [3]. Dans les pays développés, il y a eu résurgence des maladies évitables par la vaccination qu'on croyait disparues [4]. La coqueluche reste un problème de santé publique dans les pays en développement et fait l'objet d'un regain d'actualité dans les pays industrialisés [5]. Son incidence est élevée en l'absence de la vaccination [6]. Même avec la série de vaccination initiale, la recrudescence des cas est constatée chez les enfants âgés de moins de six mois, non encore immunisés ainsi que dans la tranche d’âge de 11 à 18 ans et les adultes, chez qui l'immunité a décliné [7–10]. Selon l’étude de Kandola et al. En 2005 [11], les cas de coqueluche ont chuté de 186 à 19 avec l'utilisation du vaccin à cellule complète chez l'enfant, et du vaccin acellulaire chez l'adulte. Les grands enfants servent de réservoir de transmission lorsque la couverture en rappels est faible [12]. En France, il y a eu dans les années 90 une augmentation des nouveau-nés hospitalisés pour coqueluche du fait de l'absence des rappels naturels [13]. Il est donc recommandé de faire le rappel entre 16-18 mois ainsi qu'une dose de vaccin acellulaire entre 11-13 ans [14].

En ce qui concerne la diphtérie, entre 1993-1994 il y a eu explosion d’épidémies en ex-URSS, avec plus de 290% de cas par rapport à 1992. En Russie, dans environ 4.3 fois, les enfants ayant reçu moins de quatre à cinq doses de vaccin étaient plus susceptibles de faire la diphtérie par rapport aux témoins [15,16]. Il existe dans beaucoup de pays, des problèmes liés au diagnostic, d'où l'accent mis sur le renforcement de la vaccination [17].

Quant au tétanos, des cas sont encore enregistrés chez le grand enfant. A Dakar la prévalence hospitalière était de 5,3% chez les enfants âgés de 1 à 15 ans, avec un taux de létalité de 8% [18]. En Malaisie, 22 cas ont été enregistrés à l'hôpital de Sarawak en 10 ans, la plupart survenus entre 60-90 ans [19]. De 2001–2008, 233 cas ont été enregistrés dans 45 Etats en Amérique avec une létalité de 13.2% [20]. L'immunité contre le tétanos décline avec le temps chez l'enfant vacciné, si bien qu'entre 10 et 16 ans, 1/5 d'enfant ne possède plus d'anticorps protecteurs [21]. Une immunisation contre cette maladie serait obtenue avec de doses additionnelles de vaccins combinés (diphtérie-tétanos-coqueluche) administrées dans la deuxième année de vie et à l'entrée à l’école [18].

La poliomyélite, reste endémique dans beaucoup pays, plusieurs années après le lancement de l'initiative mondiale pour son éradication. En Afrique, l'on a noté des flambées de poliomyélite en Namibie entre 1993-1999 [22], en 2006 où les adultes ont été affecté [23]. Au Cameroun, des cas ont été enregistrés en 2005, 2007 et en 2009 [24]. En 2010, sept pays ont été recontaminés, dont deux avec foyers épidémiques majeurs: le Congo-Brazzaville, le Tadjikistan, depuis lequel des cas ont été importés dans d'autres pays d'Asie Centrale [25]. Au Cameroun, l'ampleur de ces maladies évitables est sous estimée, la surveillance épidémiologique dans le cadre du PEV ne ciblant pas toutes les maladies évitables par la vaccination. Des cas passeraient inaperçus du fait des difficultés diagnostiques [26], de la faible performance de la surveillance et parce que les cas ne sont pas systématiquement déclarés au Cameroun. Ceci est d'autant plus pertinent que les résultats de l′impact épidémiologique de la vaccination dépendent de la qualité du diagnostic à l'origine des données fiables [27].

La présente étude avait pour but de déterminer le taux de rappels vaccinaux et les raisons du défaut de ces rappels chez les enfants au-delà de la cible du PEV afin de formuler des recommandations opérationnelles en faveur de la politique vaccinale au Cameroun.

## Méthodes

L’étude a eu lieu à l’école maternelle et primaire publiques de la Cité-verte et au Groupe scolaire privé Thécla tous à Yaoundé, capitale politique du Cameroun. Ces établissements étaient choisis à cause de la diversité des classes socioéconomiques de leurs élèves et de l'accessibilité géographique aisée. Le premier établissement comprenait un cycle maternel et primaire de 140 et de 800 élèves respectivement. Le second quant à lui comptait 312 et 505 élèves respectivement pour le cycle maternel et primaire.

### Définition des termes

Enfant correctement vacciné: c'est un enfant ayant reçu toute la série initiale des antigènes, à l′âge prévu par le programme de vaccination camerounais. Il s'agissait de trois doses de vaccin contre la diphtérie-tétanos-coqueluche et quatre doses de vaccin oral anti poliomyélite, une dose de vaccin anti rougeoleux et antiamaril.

Centres de vaccination agréés: ce sont des formations sanitaires publiques et privées reconnues par le gouvernement et qui sont autorisées à offrir des prestations de soins en vaccination.

### Niveau de revenu des parents

Bas: revenu moyen inférieur 40 $ US par mois; Moyen: revenu moyen supérieur à 40 $ US et inférieur à 82 $ US par mois; Élevé: revenu moyen supérieur à 82 $ US par mois.

### Type d’étude, période

Etude descriptive transversale ayant couvert la période de Septembre à Novembre 2009.

### Population d’étude

Il s'agissait d'enfants scolarisés du niveau d’éducation de base. Etaient inclus tout ceux dont l’âge variait de 2 à 7ans et dont le carnet de vaccination avait été présenté par le parent. Les élèves ont été recrutés de façon consécutive dans chacun des établissements. Nous avons sélectionné par famille un seul enfant et son parent. Le taux de rappel vaccinal chez les enfants hors cible du PEV a été extrapolé à partir du taux de 15% de rappel en DTC Polio obtenu d'une étude réalisée en 2006 chez les enfants âgés de 18 à 36 mois nés de mères séropositives au VIH [28]. En supposant que le taux de rappel vaccinal est plus faible dans la population générale (7%), il nous a fallu pour une précision de 3% inclure dans notre étude au moins 289 enfants.

### Procédure

Les données recueillies des carnets ont porté sur le nom du site, la date de la vaccination, le numéro des lots de vaccins utilisés, et les autres mentions à l'instar de la signature du prestataire de soins ou le cachet de l'institution sanitaire. Par souci d'uniformité, nous nous sommes intéressés surtout à la couverture en rappels vaccinaux contre la diphtérie, le tétanos, la coqueluche et la poliomyélite orale. Les vaccins contre l'hépatite virale B, *Haemophilus influenzae* b ayant été introduits plus tard dans le PEV respectivement en 2005 en 2009. Nous avons également mené un entretien avec les parents à la recherche des informations d'ordre sociodémographique (l’âge et la profession, le niveau d'instruction des mères et le revenu des deux parents). Chez les enfants était recherchée systématiquement la cicatrice de BCG au niveau de l'avant-bras.

### Analyse statistique

Les données ont été saisies et analysées avec le logiciel EPI INFO version 3.5.1. Les tests de chi carré et de Fisher ont été utilisés pour la comparaison des proportions. Le test était significatif pour une valeur de P<0,05.

### Considérations éthiques

Cette étude a été approuvée par le comité d’étique du ministère de la santé publique. Nous avons obtenu par ailleurs, les autorisations du ministère de l’éducation de base ainsi que celles des directeurs des établissements scolaires concernés. Le consentement écrit ou verbal des parents d’élèves étaient obtenu au préalable et la confidentialité était garantie par l'anonymat.

## Résultats

L’étude a porté sur 310 enfants dont 160 filles (51,6%) et 150 garçons (48,4%). L’âge moyen était de 4,0 ans±1,4 avec les extrêmes de 2 et 7 ans. La tranche d’âge la plus représentée était celle de 2 à 4 ans, soit 214 enfants (69%) contre 96 (31%) pour la tranche de 5 à 7ans. Les enfants vivaient dans 95,8% de cas avec leurs parents géniteurs, le reste (3,9%) avec leurs grands-parents.

### Niveau d’éducation et de revenu des parents

Toutes les mères des 310 enfants étaient scolarisées, plus de deux tiers (69%) avaient atteint le niveau d’étude secondaire, 14,9% n'avaient fait que des études primaires et 16,1% avaient le niveau supérieur. A propos du revenu, seules 3,54% mères avaient un niveau élevé, les autres étaient au niveau moyen (47,41%) ou bas (49,03%), P=0,11. La tendance était différente chez les hommes chez qui plus de 2/3 (69,67%) avaient un revenu moyen, à l'opposé 14,51% et 15,82% avaient un revenu bas ou élevé respectivement (P=0,0000).

### Statut vaccinal des enfants selon le Programme Elargi de Vaccination (PEV)

Les enfants ont été répartis selon qu'ils aient été correctement ou non correctement vaccinés. C'est ainsi que 223 enfants (71,9%) avaient été correctement vaccinés, alors que 28,1% ne l’étaient pas. L’évaluation de la couverture en BCG a montré que 308 sur 310 enfants (99,35%) avaient reçu ce vaccin à la naissance et que seuls 235 (76,3%) avaient la cicatrice de BCG à l'examen de l'avant-bras.

### Relation entre le statut vaccinal des enfants et le niveau d’éducation des mères

Le niveau d’éducation des mères avait une influence sur le statut vaccinal des enfants (Tableau 1). Plus il était élevé, mieux leurs enfants étaient correctement vaccinés (P=0,0048).

**Tableau 1:** Répartition des enfants selon leur statut en vaccins du PEV et le niveau d’éducation des mères

	**Statut vaccinal**			
	
	Correctement vaccinés		Non correctement vaccinés	
	
	**Nombre**	**%**	**Nombre**	**%**
Niveau d’éducation des mères				
Primaire	24	10,8	22	25,3
secondaire	160	71,7	54	62,1
supérieur	39	17,5	11	12,6
**Total**	223	100	87	100

### Rappels vaccinaux

Seuls les enfants qui avaient été correctement vaccinés selon le PEV ont fait des rappels, soit 29 (13%) et 24 (10,8%) pour la première dose de rappel de DTC et polio oral respectivement (Tableau 2). L'analyse des rappels vaccinaux chez les enfants pris individuellement a permis de constater que seuls 6 (2,7%) enfants sur les 223 enfants correctement vaccinés avaient également bien fait les premiers rappels DTC et Polio oral. Quant au deuxième rappel DTC et Polio oral, aucun des 96 enfants âgés de 5 à 7 ans ne l'avait reçu.

**Tableau 2:** Répartition des enfants ayant été correctement vaccinés selon le PEV en fonction du 1^er^ rappel vaccinal DTC et Polio oral

Rappels Vaccinaux	Effectif	Pourcentage
1^er^ Rappel fait		
**DTC**	29	13%
**Polio**	24	10,8%
1^er^ Rappel non Fait	170	76,2%
Total	223	100%

### Caractéristiques sociodémographiques des parents des enfants ayant reçu le 1^er^ rappel vaccinal DTC-Polio oral

Le Tableau 3 montre que plus le niveau d'instruction et le revenu des mères était élevés, d'avantage les enfants avaient eu le premier rappel vaccinal (P=0,0000). Le revenu du père quant à lui n'a pas influencé sensiblement les rappels vaccinaux (P=0,051).

**Tableau 3:** Répartition des enfants ayant reçu le premier rappel vaccinal selon le niveau d’éducation et le revenu des parents

**Caractéristiques parentales**	Nombre	OR	IC	P
**Niveau d’éducation des mères**				
Primaire	0	0	-	0,0054
Secondaire	2	0,18	0,03-1	
Supérieur	4	10,4	1,83-58	
**Revenu des mères**				
Bas	0	0	-	0,0000
Moyen	3	1,14	0,22-5	
Elevé	3	30	5,11-17	
**Revenu des pères**				
Bas	1	1,4	0,15-12	0,051
Moyen	2	0,18	0,03-1,05	
Elevé	3	5,75	1,11-29	

OR: Odd Ratio, IC: Intervalle de Confiance a 95%

### Les raisons de non administration des premières doses de rappel vaccinal

**Figure 1:**
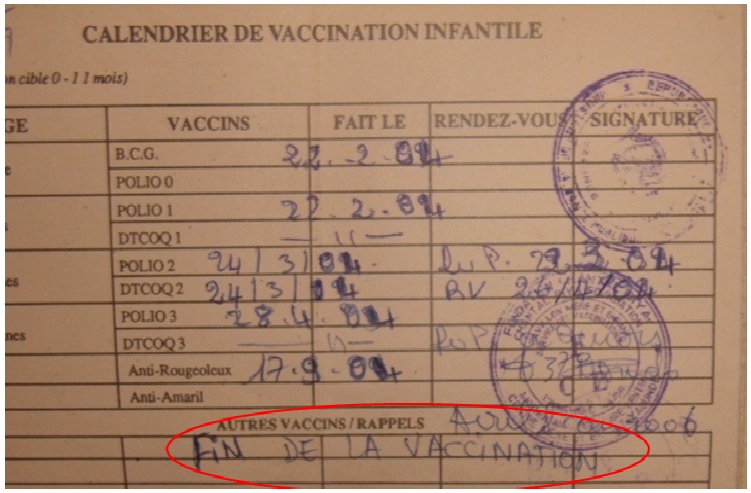
Image d'un carnet de vaccination portant la mention Fin de la vaccination chez un enfant ayant terminé la première série de vaccination

Les dates exactes de rendez-vous pour le rappel étaient précisées dans les carnets de vaccination de six enfants correctement vaccinés et ayant fait le 1er rappel vaccinal DTC et Polio. Seuls deux parents avaient été sensibilisés verbalement par le personnel de santé. Les principaux motifs de non administration des rappels vaccinaux étaient l'ignorance (50% des mères), le prix élevé des vaccins (48,69%). En effet, il nous a été rapporté que certains centres de vaccination faisaient payer le vaccin polio oral. Par ailleurs, la désinformation par le personnel de la vaccination était la troisième raison de non rappel; sur trois carnets était portée la mention “fin de la vaccination” après les vaccins du PEV (Figure 1).

### Les autres problèmes identifiés sur les carnets de vaccination

Les autres irrégularités identifiées dans les carnets de vaccinations étaient l'absence de la signature du prestataire de soins, de la date de vaccination, ou du lieu où a été administré le vaccin. Tous les rappels vaccinaux n'ont pas été faits dans les centres de vaccination agrées, c'est ainsi que douze enfants ont reçu le 1er rappel DTC et Polio oral dans des centres inconnus.

## Discussion

Le découpage en tranches d’âge de 2-4 ans et 5-7 ans dans cette étude correspond aux âges auxquels les enfants sont supposés avoir reçu déjà la série de vaccination primaire et les premiers et deuxièmes rappels vaccinaux. Dans la présente étude, 223 enfants (71,90%) étaient correctement vaccinés, ce taux est en deçà de l'objectif national de la couverture fixée à 88% en 2009 au Cameroun pour l'antigène traceur DTC3 [29]. D'après une étude menée à Chicago aux Etats Unis, seul 76.6% d'enfant avaient complété leur série de vaccination avant l'entrée à l’école [30]. A Conakry 19% d'enfants avaient eu plusieurs contacts avec les services curatifs et préventifs, mais n'ont pas reçu leurs vaccins à ces occasions [31].

Les établissements scolaires joueraient un rôle crucial dans la promotion de la vaccination par l’éducation des familles et des communautés [32]. Au Cameroun, les carnets de vaccination sont exigés à l'admission dans les sections maternelles des établissements scolaires de base. Ces carnets ne sont pas malheureusement exploités pour des informations pertinentes sur l’état vaccinal des enfants. Il serait important que soient saisies, ces opportunités pour rattraper les enfants qui ont raté les doses de leur vaccin. Dans la présente étude, beaucoup de parents ne possédaient pas ces carnets, il se pourrait qu'ils ne soient jamais mis à leur disposition. Pourtant des études ont montré que la dotation des enfants en cet outil contribue à améliorer leur couverture vaccinale [33,34]. Au Sénégal, ces interventions ont entraîné une couverture vaccinale élevée (jusqu’à 80%), mais non durable. Après leur arrêt, la couverture vaccinale a chuté en-dessous de 40% [35]. Au Pakistan, l'amélioration de la disponibilité des carnets de vaccination, seule ou en association avec la sensibilisation des parents a contribué significativement à l'augmentation du suivi de la vaccination des enfants [36].

Certains facteurs sociaux influenceraient la vaccination des enfants, il est ressorti de la présente étude que, le niveau élevé d'instruction et du revenu des mères a eu un effet positif sur le statut vaccinal de leurs enfants. Bien qu'au Burkina Faso, le niveau d’éducation n'ait pas affecté la vaccination complète [37], plusieurs études ont montré que les enfants des mères ayant un niveau d'instruction élevé étaient vaccinés complètement contrairement à ceux dont les mères n'avaient pas été instruites [34,38,39]. De même, les enfants dont les pères n'avaient pas atteint le niveau d’étude secondaire étaient 2,3 fois moins vaccinés que ceux dont le père étaient plus instruits. Dans notre étude, le niveau d'instruction des pères n'a pas été évalué, par contre leur revenu n'a pas influencé la vaccination des enfants.

En ce qui concerne les rappels vaccinaux (DTC et Polio oral), le taux était extrêmement faible et aucun enfant n'avait bénéficié de la deuxième dose de rappel. En Catalogne (Espagne), une étude a montré que l'effet protecteur du vaccin coquelucheux était de courte durée, les dose de rappels régulières chez les adolescents et /ou les adultes permettraient de diminuer l'incidence de la maladie [40]. En matière de prévention des maladies, certains pays de la région OMS ont inscrit les rappels vaccinaux dans leur programme. L'académie américaine de pédiatrie recommande que le vaccin acellulaire anticoquelucheux combiné à l'anatoxine diphtérique et tétanique soit administré aux enfants entre 11 et 12 ans pour prévenir la coqueluche chez les adolescents [7].

Au Cameroun, la persistance des anticorps anti tétanique serait limitée, du fait de l'immaturité immunologique chez le très jeune enfant. Une étude sur la réactivation immunologique a permis aux auteurs de conclure que l'administration des doses de rappel permettrait de maintenir la protection durable contre le tétanos [41].

Par rapport à la politique vaccinale, l'OMS recommande que les pays qui ont atteint une couverture élevée (90%) pour la série de vaccination primaire, fassent une ou deux doses de rappel [42]. Dans les pays à ressources limitées, les rappels sont relégués au second plan tant que la couverture vaccinale n'excède pas 90%. Cette recommandation de l'OMS ne serait pas pour l'instant applicable au Cameroun, car la couverture pour les vaccins du PEV reste problématique. La subvention par l’état des doses de rappels a été suspendue par ailleurs, du fait de la dégradation de la situation financière. Au-delà de 11 mois révolus, les vaccins (doses de rattrapage et de rappels) sont à la charge des parents. Seules les activités supplémentaires de vaccination (campagnes de masse et semaines d'action de santé et nutrition maternelle et infantile) offrent gratuitement les occasions pour les rattrapages vaccinaux chez les enfants de lus de 11 mois.

Il a été dé montré une corrélation entre le statut des enfants complètement vaccinés et le niveau de connaissance des parents en matière de la vaccination [43,44]. La communication en santé jouerait un rôle important dans les programmes de vaccination. [45,46]. En dehors des actions de sensibilisation de proximité faites par une minorité de professionnels de santé dans certaines grandes métropoles du pays, les rappels restent peu connus des populations. Il faut noter également, le faible engagement politique dans la formation du personnel en matière de vaccination et l'insuffisance de communication et de mobilisation sociale. Le système entretiendrait ainsi, les occasions manquées par la non vaccination en routine des enfants, qui n'ont pas pu avoir toutes les doses de vaccin avant leur premier anniversaire. La stratégie de la Prise en Charge Intégrée des Maladies de l'Enfant, offre une opportunité pour les rattrapages avec les doses restantes des ces vaccins [47], mais sa mise en æuvre effective pose encore des problèmes au Cameroun. Contrairement à d'autres études [48], aucun parent n'avait évoqué l'effet secondaire de la vaccination comme motif de non administration de rappel. L'attitude, les croyances et le comportement des parents interrogés n’étaient pas contre la vaccination.

Alors qu'au Cameroun le vaccin polio oral est gratuit chez tous les enfants, très peu de personnel de santé le recommandent en dehors de la cible du PEV. Contrairement aux autres vaccins, les rappels et les doses de rattrapage au-delà de 12 mois seraient inaccessibles financièrement à la plupart des familles, une dose de vaccin DTC coûtant environ 12 $ US. Cet obstacle a été l'une des raisons de non administration des doses de rappel vaccinal du fait du coût élevé du payement direct qu'il induirait. Cet aspect financier est à relativiser car du point de vue social, les enfants étaient gardés au sein de leur famille, l'on qualifierait cet environnement de favorable puisque certains parents ont pu inscrire leurs enfants dans un établissement privé où les droits scolaires sont très élevés. Des études ont montré la différence de couverture vaccinale entre les enfants non assurés issus des parents pauvres et ceux ayant une assurance privée [49,50]. La mise en place au Cameroun, d'un système d'assurance maladies garantirait l’équité dans l'offre des vaccins en dehors de la routine.

### Limites de l’étude

Le statut vaccinal des enfants serait mal estimé dans cette d’étude, les carnets de vaccination n’étaient pas disponibles chez tous les enfants. La couverture en rappel serait différente, si tous les carnets de vaccination étaient exploités. Certains parents se seraient procurés des vaccins en dehors de centres agrées et par conséquent le vaccin administré n'aurait pas été reporté dans les carnets des enfants. Par ailleurs le biais de mémoire a été évité par l’évaluation de la couverture en rappel à partir des carnets de vaccination. Le site d’étude s'est limité à deux établissements de Yaoundé une zone urbaine qui n'est donc pas représentative de la population générale. La petite taille de l’échantillon constituerait aussi un biais important. L’étude n'a pas évalué la couverture en vaccin contre l'hépatite B et les infections à *Haemophilus influenzae b*. Ces antigènes n'ont été introduits que 5 ans et un an auparavant respectivement pour l'hépatite B et l'Haemophilus.

## Conclusion

Le niveau de couverture vaccinale dans notre population d’étude était en dessous de l'objectif de couverture nationale. Le taux de rappel était encore plus faible pour le premier rappel et nul pour le second. Le manque d'information des parents ainsi que les obstacles financiers constitueraient un frein important à l'immunisation des enfants au-delà de 12 mois. L'amélioration du taux de rappel vaccinal passerait par un engagement politique vis-à-vis du renforcement des connaissances en matière de la vaccination, du personnel de santé en charge des enfants. L'appui du gouvernement permettrait d'améliorer l'accessibilité financière à ces vaccins de rappel. Par ailleurs, le renforcement de la communication avec les parents et leur sensibilisation à chaque contact avec une formation sanitaire est une nécessité. Les informations sur le calendrier vaccinal et les modalités y compris pour les rappels (gratuité du PEV, le prix des vaccins de rappel et centres agréés de vaccination) doivent être données aux parents dès la naissance de leur enfant. Les responsables des établissements de base ne doivent plus se contenter simplement à s'assurer de la disponibilité des livrets de vaccination au moment de l'inscription des enfants. Ils doivent s'impliquer dans la détection des enfants devant bénéficier du rattrapage et des rappels vaccinaux. Des efforts en matière d’éducation de la jeune fille, future mère élèverait leur niveau d'adhésion au programme, car la santé de leurs enfants en dépendra. Enfin, la mise en æuvre de la surveillance fournirait des données épidémiologiques sur les maladies évitables par la vaccination, outil indispensable pour influencer la décision politique sur les rappels vaccinaux.

## Remerciements

Nous adressons nos sincères remerciements aux responsables des deux établissements d’éducation de base ainsi qu'aux enseignants. Nous remercions aussi, l’épidémiologiste pour les aspects statistiques du document, et le secrétaire qui a saisi le document.

## References

[1] WHO Programme élargi de vaccination.

[2] Pichichero ME (2009). Booster vaccinations: can immunologic memory outpace disease pathogenesis?. Pediatrics.

[3] Vincent-Ballereau F, Schrive I, Fisch A, Laurichesse H, Romasko C, Baron D, Dublanchet A, Deteix P, Tournade S, Rey I (1995). Immunité antidiphtérique de la population Française adulte d'après une enquête sérologique multicentrique. Bulletin Epidémiologique hebdomadaire.

[4] Fung KSC, Yeung WL, Wong TW, So KW, Cheng AFB (2004). Pertussis-a re-emerging infection?. Journal of Infection.

[5] Baron Sabine, Bégué Pierre, Grimprel Emmanuel (1994). Epidémiologie de la coqueluche dans les pays industrialisés. Cahier santé.

[6] Préziosi Marie-Pierre, Yam Abdoulaye, Wassilak Steven G F, Chabirand Laurence, Simaga Aminata, Ndiaye Malick, Dia Marème, Dabis François, Simondon François (2002). Epidemiology of pertussis in a West African community before and after introduction of a widespread vaccination program. Am J Epidemiol.

[7] Greenberg, David P (2005). Pertussis in adolescents: increasing incidence brings attention to the need for booster immunization of adolescents. Pediatric Infectious Disease Journal.

[8] Brennan Muireann, Strebel Peter, George Harvey, Yih W Katherine, Tachdjian Raffi, Lett Susan M, Cassiday Pam, Sanden Gary, Wharton Melinda (2000). Evidence for transmission of pertussis in schools, Massachusetts, 1996: epidemiologic data supported by pulsed-field gel electrophoresis studies. The Journal of Infectious Diseases.

[9] Crespo Inma, Cardeñosa Neus, Godoy Pere, Carmona Gloria, Sala M Rosa, Barrabeig Irene, Alvarez Josep, Minguel Sofia, Camps Neus, Caylà Joan, Batalla Joan, Codina Gemma, Dominguez Angela (2011). Epidemiology of pertussis in a country with high vaccination coverage. Vaccine.

[10] Yih W Katherine, Lett Susan M, des Vignes Franka N, Garrison Krista M, Sipe, Patricia L, Marchant Colin D (2000). The Increasing Incidence of Pertussis in Massachusetts Adolescents and Adults 1989–1998. The Journal of Infectious Diseases.

[11] Kandola Kami, Lea Amy, White Wanda, Santos Maria (2005). A comparison of pertussis rates in the Northwest Territories: Pre- and postacellular pertussis vaccine introduction in children and adolescents. Can J Infect Dis Med Microbiol.

[12] Grimprel Emmanuel, Baron Sabine, Lévy-Bruhl Daniel, Garnier Jean Marc, N'jamkepo Elisabeth, Guiso Nicole, Bégué Pierre (1999). Influence of vaccination coverage on pertussis transmission in France. Lancet.

[13] Guiso Nicole (2007). Impact de la vaccination sur l’épidémiologie des maladies infectieuses- Exemple de la coqueluche. Med Sci (Paris).

[14] CDC (2006). Preventing Tetanus, Diphtheria, and Pertussis Among Adolescents: Use of Tetanus Toxoid, Reduced Diphtheria Toxoid and Acellular Pertussis Vaccines Recommendations of the Advisory Committee on Immunization Practices (ACIP). Morbidity and Mortality Weekly Report.

[15] Bisgard Kristine M, Rhodes Philip, Hardy Iain RB, Litkina Irina L, Filatov Nikolai N, Monisov Anatoli A, Wharton Melinda (2000). Diphtheria Toxoid Vaccine Effectiveness: A Case-Control Study in Russia. The Journal of Infectious Diseases.

[16] Vitek Charles R, Brennan Muireann B, Gotway Carol A, Bragina Vera Y, Govorukina Nadezhda V, Kravtsova Olga N, Rhodes Philip H, Bisgard Kristine M, Strebel Peter M (1999). Risk of diphtheria among schoolchildren in the Russian Federation in relation to time since last vaccination. Lancet.

[17] Golaz Anne, Hardy Iain R, Strebel Peter, Bisgard Kristine M, Vitek Charles, Popovic Tanja, Wharton Melinda (2000). Epidemic Diphtheria in the Newly Independent States of the Former Soviet Union: Implications for Diphtheria Control in the United States. The Journal of Infectious Diseases.

[18] Soumaré M, Seydi M, Ndour CT, Ndour JD, Diop BM (2005). Aspects épidémiologiques, cliniques et pronostiques du tétanos juvénile à Dakar. Sénégal. Bull Soc Pathol Exot.

[19] Lau LG, Kong KO, Chew PH (2001). Ten-year retrospective study of tetanus at a general hospital in Malaysia. Singapore Med J.

[20] Tetanus Surveillance-United States CDC (2001). 2008. MMWR Morb Mortal Wkly Rep. 2011 Apr ;60.

[21] Gergen Peter J, Mcquillan Geraldine M, Kiely Michele, Ezzati-Rice Trena M, Sutter Roland W, Virella Gabriel (1995). A population-based serologic survey of immunity to tetanus in the United States. N Engl J Med.

[22] Gaspar Miguel, Morals Alda, Brumana Luisa, Stella Alberto A (2000). Outbreak of poliomyelitis in Angola. J Infect Dis.

[23] OMS (2006). Flambée de poliovirus sauvage de type 1 chez des adultes. Namibie, 2006.Wkly Epidemiol Rec.

[24] OMS (2011). Mise à jour hebdomadaire sur l'Initiative d'Eradication de la Polio en Afrique Centrale. http://www.polioeradication.org/Portals/0/Document/AboutUs/Governance/IMB/deliberations/CentralAfrica_Weekly_polio_updates_20110322.pdf.

[25] Antona Denise et Guérin Nicole (2010). L’éradication de la poliomyélite: ou en est-on en 2010?. Bulletin épidémiologique hebdomadaire.

[26] Hochwald Ori, Bamberger Ellen, Srugo Isaac (2006). The return of pertussis: who is responsible? What can be done?. Isr Med Assoc J.

[27] De Carvalho Aroldo P, Pereira Eliane Mara Cesário (2006). Acellular pertussis vaccine for adolescents. J Pediatr (Rio J).

[28] Tejiokem MC, Gouandjika I, Béniguel L, Endegue Zanga MC, Tene G, Gody JC, Njamkepo E, Kfutwah A, penda I, Bilong C, Rousset D, Pouillot R, Tangy F, Baril L (2007). HIV-infected children living in Central Africa have low persistence of antibodies to vaccines used in the expanded program on immunization. PLoS One.

[29] Ministère de la santé publique Cameroun (2008). Plan Pluriannuel Complet 2007-2011 du Programme Elargi de Vaccination.

[30] Dominguez Samuel R, Parrott J Scott, Lauderdale Diane S, Daum Robert S (2004). On-time immunization rates among children who enter Chicago public school. Pediatrics.

[31] Cutts FT, Zell ER, Soares AC, Diallo S (2000). Obstacles to Achieving Immunization for all : missed immunization opportunities and inappropriately timed immunization. Journal of Tropical Pediatrics. ;37.

[32] Megan C Lindley, Lynda Boyer-Chu, Daniel B Fishbein, Maureen Kolasa, Amy B Middleman, Thad Wilson, JoEllen Wolicki, Susan Wooley, the Working Group (2008). The role of schools in strengthening delivery of new adolescent vaccinations. Pediatrics.

[33] Rosenthal Jorge, Rodewald Lance, McCauley Mary, Berman Stephen, Irigoyen Matilde, Sawyer Mark, Yusuf Hussein, Davis Ronald, Kalton Graham (2004). Immunization coverage levels among 19- to 35-month-old children in 4 diverse, medically underserved areas of the united states. Pediatrics.

[34] McElligott James T, Darden Paul M (2010). Are patient-held vaccination records associated with improved vaccination coverage rates?. Pediatrics. ; :ee472.

[35] Chippaux JP, Marra A, Diallo A, Simondon F, Etard JF (1984). Analyse de l’évolution de la couverture vaccinale à Niakhar, région rurale du Sénégal, entre et 2003. Bull Soc Pathol Exot. ; 99.

[36] Usman Hussain R, Rahbar Mohammad H, Kristensen Sibylle, Vermund Sten H, Kirby Russell S, Habib Faiza, Chamot Eric (2011). Randomized controlled trial to improve childhood immunization adherence in rural Pakistan: redesigned immunization card and maternal education. Trop Med Int Health.

[37] Sia Drissa, Fournier Pierre, Kobiané Jean-François, Sondo Blaise K (2009). Rates of coverage and determinants of complete vaccination of children in rural areas of Burkina Faso (1998-2003). BMC Public Health.

[38] Torun Sebahat D, Bak?rc? Nadi (2006). Vaccination coverage and reasons for non-vaccination in a district of Istanbul. BMC Public Health.

[39] Luman Elizabeth T, McCauley Mary Mason, Shefer Abigail, Chu Susan Y (2003). Maternal characteristics associated with vaccination of young children. Pediatrics.

[40] Cardeñosa Neus, Romero Marcos, Quesada Mariela, Oviedo Manuel, Carmona Gloria, Codina Gemma, Jansà Josep M, Domínguez Angela (2009). The Pertussis Working Group of Catalonia. Is the vaccination coverage established enough to control pertussis, or it is a re-emerging disease?. Vaccine.

[41] Posfay-Barbe Klara M, Kobela Marie, Sottas Cedric, Grillet Stéphane, Taguebue Jean, Tetanye Ekoe, Lambert Paul Henri, Lecoultred Claude, Siegrist Claire Anne (2010). Frequent failure of adolescent booster responses to tetanus toxoid despite infant immunization: Waning of infancy-induced immune memory?. Vaccine.

[42] WHO Global Program for Vaccines and Immunization 1995. Immunization policy.

[43] Babatsikou Fotoula, Vorou Rengina, Vardaki Zambia, Galani Stavroula, Ktenas Eftichios, Koutis Charilaos (2010). Childhood vaccination uptake and factors affecting this in Athens. Greece, Health Science Journal.

[44] Sanou Aboubakary, Simboro Seraphin, Kouyaté Bocar, Dugas Marylène, Graham Janice, Bibeau Gilles (2009). Assessment of factors associated with complete immunization coverage in children aged 12-23 months: a cross-sectional study in Nouna district, Burkina Faso. BMC International Health and Human Rights.

[45] Porter Robert W, Steinglass Robert, Kaiser Javaid, Olkhovsky Paul, Rasmuson Mark, Dzhatdoeva Fatima A (2000). Fishman Boris and Bragina Vera. Role of health communications in Russia's diphtheria immunization program. The Journal of Infectious Diseases.

[46] Owais Aatekah, Hanif Beenish, Siddiqui Amna R, Agha Ajmal, Zaidi Anita KM (2011). Does improving maternal knowledge of vaccines impact infant immunization rates? A communitybased randomized-controlled trial in Karachi, Pakistan. BMC Public Health.

[47] Ministère de la Santé Publique Cameroun (2005). Normes et Standards du programme élargi de vaccination.

[48] Gust Deborah A, Strine Tara W, Maurice Emmanuel, Smith Philip, Yusuf Hussain, Wilkinson Marilyn, Battaglia Michael, Wright Robert, Schwartz Benjamin (2004). Under immunization among children: effects of vaccine safety concerns on immunization status. Pediatrics.

[49] Santoli Jeanne M, Huet Natalie J, Smith Philip J, Barker Lawrence E, Rodewald Lance E, Inkelas Moira, Olson Lynn M, Halfon Neal (2004). Insurance status and vaccination coverage among us preschool children. Pediatrics.

[50] Molinari Noëlle-Angélique M, Kolasa Maureen, Messonnier Mark L, Richard A Schieber (2007). Out-of-pocket costs of childhood immunizations: A comparison by type of insurance plan. Pediatrics.

